# Revisiting the STEC Testing Approach: Using *espK* and *espV* to Make Enterohemorrhagic *Escherichia coli* (EHEC) Detection More Reliable in Beef

**DOI:** 10.3389/fmicb.2016.00001

**Published:** 2016-01-22

**Authors:** Sabine Delannoy, Byron D. Chaves, Sarah A. Ison, Hattie E. Webb, Lothar Beutin, José Delaval, Isabelle Billet, Patrick Fach

**Affiliations:** ^1^Food Safety Laboratory, Université Paris-Est, Anses (French Agency for Food, Environmental and Occupational Health and Safety), Platform IdentyPathMaisons-Alfort, France; ^2^Department of Animal and Food Sciences, Texas Tech UniversityLubbock, TX, USA; ^3^Division of Microbial Toxins, National Reference Laboratory for Escherichia coli, Federal Institute for Risk AssessmentBerlin, Germany; ^4^Laboratoire de Touraine, (LDA37) Conseil DépartementalTours, France; ^5^SAS Charal Groupe BigardCholet, France

**Keywords:** STEC, O157, non-O157, *espK*, *espV*, *ureD*, *Z2098*, CRISPR

## Abstract

Current methods for screening Enterohemorrhagic *Escherichia coli* (EHEC) O157 and non-O157 in beef enrichments typically rely on the molecular detection of *stx, eae*, and serogroup-specific *wzx* or *wzy* gene fragments. As these genetic markers can also be found in some non-EHEC strains, a number of “false positive” results are obtained. Here, we explore the suitability of five novel molecular markers, *espK, espV, ureD, Z2098*, and CRISPR_O26:H11_ as candidates for a more accurate screening of EHEC strains of greater clinical significance in industrialized countries. Of the 1739 beef enrichments tested, 180 were positive for both *stx* and *eae* genes. Ninety (50%) of these tested negative for *espK, espV, ureD*, and *Z2098*, but 12 out of these negative samples were positive for the CRISPR_O26:H11_ gene marker specific for a newly emerging virulent EHEC O26:H11 French clone. We show that screening for *stx, eae, espK*, and *espV*, in association with the CRISPR_O26:H11_ marker is a better approach to narrow down the EHEC screening step in beef enrichments. The number of potentially positive samples was reduced by 48.88% by means of this alternative strategy compared to the European and American reference methods, thus substantially improving the discriminatory power of EHEC screening systems. This approach is in line with the EFSA (European Food Safety Authority) opinion on pathogenic STEC published in 2013.

## Introduction

Shiga toxin-producing *Escherichia coli* (STEC) are important zoonotic pathogens comprising more than 400 serotypes (Beutin and Fach, 2014). A fraction of these serotypes are able to cause bloody diarrhea and may progress to hemolytic uremic syndrome (HUS). This subset of STECs is termed Enterohemorrhagic *E. coli* (EHEC) (Beutin and Fach, 2014). STEC O157:H7 has been the first pathogenic *E. coli* whose presence in foodstuffs was regulated. Today, non-O157 STEC infections have increased greatly, sometimes accounting for up to 70% of notified STEC infections (Brooks et al., 2005; Johnson et al., 2006; Gould et al., 2013; EFSA, 2014). Consequently, regulations in the US and in the EU have evolved to include some non-O157 STEC serogroups together with serotype O157:H7.

Successful implementation of these regulations in the food industry across the world requires effective detection methods that are both specific and sensitive. Detection of non-O157 STEC in foods is particularly challenging because these bacteria lack phenotypic characteristics that distinguish them invariably from the large number of non-STEC flora that share the same habitat. Additionally, they may be present in very low numbers, with a heterogeneous distribution in the food matrices. The environment of the food matrix may also trigger stress responses from the STEC strains and induce latent physiological states that further complicate detection (Wang et al., 2013).

In the absence of a clear definition of virulent STEC strains, the ISO/CEN 13136:2012 Technical Specification (ISO, 2012) and US MLG5B.05 (USDA-FSIS, 2014) use a stepwise approach, comprising an initial screening step for virulence genes (Shiga toxin genes, *stx*, and intimin gene, *eae*), followed by testing of O-serogroup specific gene markers. Because the *stx* and *eae* genes can be independently present in a number of non-pathogenic strains of *E. coli* and other *Enterobacteriaceae*, the first screening step generates numerous signals from samples that do not necessarily contain a true EHEC strain. These *stx*/*eae* positive enrichments must then be subjected to a second screening targeting the O-group gene markers. As testing is completed on enrichment broths that contain a mixture of different cells, the different target gene signals (i.e., *stx, eae*, and the O-group markers) may arise from different individual non-pathogenic strains. Lastly it is necessary to perform an isolation step to confirm the presence of the different markers in a single isolate by PCR.

We have previously shown that combining the detection of *espK* with either *espV, ureD*, or *Z2098* is a highly sensitive and specific approach for identifying the top seven clinically important EHEC serotypes in industrialized countries (Delannoy et al., 2013a). These markers were shown to be preferentially associated with *E. coli* strains carrying *stx* and *eae* genes, known as typical EHEC, and could be used in conjunction with *stx*/*eae* screening to better identify samples that may be more likely to contain a true EHEC.

Recently, a new clone of STEC O26:H11 harboring *stx2a* and strongly associated with HUS has emerged (Bielaszewska et al., 2013). Characterization of O26:H11 *stx2* circulating in France (Delannoy et al., 2015a,b) demonstrated that some *stx2a* or *stx2d* positive strains do not have any of the *espK, espV, ureD*, or *Z2098* markers. Hence, such clones would evade a first detection step solely based on *stx*/*eae* and a combination of those genetic markers. Therefore, we identified a CRISPR (Clustered Regularly Interspaced Short Palindromic Repeats) sequence specific for this O26:H11 EHEC French clone (Delannoy et al., 2015a). Detection of this CRISPR sequence would advantageously be combined with *stx* and *eae* in the first screening step to identify this new O26:H11 clone. Furthermore, we have previously developed CRISPR-based real-time PCR assays able to detect the top seven EHEC serotypes and the German O104:H4 STEC clone responsible for a very large European STEC outbreak in 2011 (Delannoy et al., 2012a,b; Miko et al., 2013). These CRISPR PCR assays proved highly sensitive and specific when tested on a large collection of *E. coli* strains comprising various *E. coli* pathogroups (Delannoy et al., 2012a,b). Such CRISPR markers may substitute O group testing in a more targeted second step.

Different approaches have been tested to refine the detection systems for EHEC. Some involve detection of *ecf1*, a plasmid gene highly associated with *E. coli* strains that are positive for *stx, eae*, and *ehxA* (Livezey et al., 2015); while others involve detection of specific *eae*-subtypes (Bugarel et al., 2010). The top seven EHEC serotypes are exclusively associated with certain *eae*-subtypes (Oswald et al., 2000; Bugarel et al., 2010; Madic et al., 2010). Indeed, O157:H7 and O145:H28 serotypes are associated with the *eae*-gamma subtype; O26:H11 are associated with the *eae*-beta subtype; O103:H2, O121:H19, and O45:H2 are associated with the *eae*-epsilon subtype; and O111:H8 are associated with the *eae*-theta subtype. Real-time PCR targeting these *eae*-subtypes were previously developed and tested in raw milk cheese (Madic et al., 2011) and cattle feces (Bibbal et al., 2014). These could also be used as targets in a more targeted second step.

We attempted to develop an alternative real-time PCR-based approach to improve the detection of the clinically important EHEC by reducing the number of potential positive samples that require further confirmation of the O-antigen markers. We also aimed at reducing the number of samples for which isolation is attempted, as the isolation step is laborious, time-consuming, and not always successful (Wang et al., 2013). The objective of this project was to evaluate the discriminatory power of these various genetic markers to predict the presence of the top seven EHEC serogroups compared to that of the strategy proposed by the ISO/CEN TS13136:2012 (ISO, 2012) and MLG5B.05 (USDA-FSIS, 2014).

## Materials and methods

### *E. coli* control strains

*E. coli* control strains used for this study (Table 1) comprised a panel of ATCC *E. coli* strains (*n* = 42) and *E. coli* reference strains derived from the BfR and Anses collections (*n* = 13). The origin and characteristics of the *E. coli* strains from BfR and Anses have been previously described (Delannoy et al., 2012a, 2013a). Cultivation of bacteria and preparation of DNA was performed as previously described (Delannoy et al., 2012a, 2013a).

**Table 1:** **PCR-screening of the genetic markers in STEC isolates used as reference**.

**Strain**	**Code**	**Serotype**	***stx1***	***stx2***	***eae***	***eae*-beta**	***eae*-gamma**	***eae*-epsilon**	***eae*-theta**	***espK***	***espV***	***Z2098***	***ureD***	**CRISPR_O26:H11_ SP_O26-E**
ATCC-2129	TW07865	O145:H28	−	+	+	−	+	−	−	+	+	+	+	−
ATCC-2192	99–3311	O145	+	+	+	−	+	−	−	+	+	+	+	−
ATCC-2194	2001–3022	O145	−	+	+	−	+	−	−	+	+	+	+	−
ATCC-2195	2002–3034	O145	+	−	+	−	+	−	−	+	+	+	+	−
ATCC-2206	2000–3413	O145	−	+	+	−	+	−	−	+	+	+	+	−
ATCC-2208	2003–3054	O145	−	+	+	−	+	−	−	+	+	+	+	−
ATCC-2181	00–3412	O26:H11	+	−	+	+	−	−	−	+	+	+	+	−
ATCC-2183	2011–3139	O26:H11	+	−	+	+	−	−	−	+	+	+	+	−
ATCC-2186	99–3294	O26:H11	+	−	+	+	−	−	−	+	+	+	+	−
ATCC-2188	99–3301	O26:H11	+	−	+	+	−	−	−	+	+	+	+	−
ATCC-2196	2003–3014	O26:H11	+	+	+	+	−	−	−	+	−	+	+	−
ATCC-2204	2001–3234	O26:H11	+	−	+	+	−	−	−	+	+	+	+	−
ATCC-2205	2003–3023	O26:H11	+	−	+	+	−	−	−	+	+	+	+	−
ATCC-2442	ATCC-2442	O26	+	−	+	+	−	−	−	+	−	+	+	−
CB14699	CB14699	O26:H11	−	+	+	+	−	−	−	−	−	−	−	+
ATCC-2187	99–3300	O121:H19	−	+	+	−	−	+	−	+	+	+	+	−
ATCC-2203	2000–3370	O121:H19	−	+	+	−	−	+	−	+	+	+	+	−
ATCC-2219	2002–3211	O121:H19	−	+	+	−	−	+	−	+	+	+	+	−
ATCC-2220	10C–3041	O121:H19	−	+	+	−	−	+	−	+	+	+	+	−
ATCC-2221	09C–3857	O121:H19	+	+	+	−	−	+	−	+	+	+	+	−
ATCC-43890	CDC–C984	O157:H7	+	−	+	−	+	−	−	−	+	+	+	−
ATCC-43895	EDL933	O157:H7	+	+	+	−	+	−	−	+	+	+	+	−
ATCC-179	CDC1997–3215	O111:H8	+	+	+	−	−	−	+	+	+	+	+	−
ATCC-181	CDC1999–3249	O111:H8	+	+	+	−	−	−	+	+	+	+	+	−
ATCC-184	CDC2000–3025	O111:H8	+	−	+	−	−	−	+	+	+	+	+	−
ATCC-2180	00–3237	O111:H8	+	+	+	−	−	−	+	+	+	+	+	−
ATCC-2201	2002–3092	O111:H8	+	−	+	−	−	−	+	+	+	+	+	−
ATCC-2182	2001–3010	O111	+	−	+	−	−	−	+	+	+	+	+	−
ATCC-2209	2001–3357	O111	+	+	+	−	−	−	+	+	+	+	+	−
ATCC-2440	ATCC-2440	O111	+	+	+	−	−	−	+	+	+	+	+	−
ATCC-2441	ATCC-2441	O111	+	−	+	−	−	−	+	+	+	+	+	−
ATCC-2202	99–3075	O45:H2	+	−	+	−	−	+	−	+	−	+	+	−
ATCC-2185	99–3291	O45:H2	+	−	+	−	−	+	−	+	−	+	+	−
ATCC-2189	99–3303	O45:H2	+	−	+	−	−	+	−	+	−	+	+	−
ATCC-2191	98–3167	O45:H2	+	−	+	−	−	+	−	+	−	+	+	−
ATCC-2193	2000–3039	O45:H2	+	−	+	−	−	+	−	+	−	+	+	−
ATCC-2198	98–3215	O45:H2	+	−	+	−	−	+	−	+	−	+	+	−
ATCC-2200	2001–3225	O103:H11	+	−	+	+	−	−	−	+	+	+	+	−
ATCC-2215	2006–3008	O103:H11	+	−	+	−	−	+	−	+	+	+	+	−
ATCC-2207	2001–3304	O103:H2	+	−	+	−	−	+	−	+	+	+	+	−
ATCC-2210	2003–3112	O103:H2	+	−	+	−	−	+	−	+	+	+	+	−
PMK5	PMK5	O103:H2	+	−	+	−	−	+	−	+	+	+	−	−
CB12062	CB12062	O103:H2	+	−	+	−	−	+	−	+	+	+	−	−
CB12092	CB12092	O103:H2	+	−	+	−	−	+	−	+	+	+	−	−
CB11097	CB11097	O103:H25	−	+	+	−	−	−	+	+	+	+	+	−
ATCC-2213	2005–3546	O103:H25	+	−	+	−	−	−	+	+	+	+	+	−
ATCC-2199	2000–3281	O103:H25	+	−	+	−	−	−	+	+	+	+	+	−
CB11784	ECA34	O5	+	−	+	+	−	−	−	+	−	+	+	−
CB12339	CB12339	O5	+	−	+	+	−	−	−	+	−	+	+	−
CB13683	5906	O55:H7	−	+	+	−	+	−	−	+	+	−	−	−
7123A	7123A	O55:H7	−	+	+	−	+	−	−	+	+	−	−	−
CB7035	CB7035	O118:H16	+	+	+	+	−	−	−	+	−	+	+	−
CB8255	CB8255	O118:H16	+	−	+	+	−	−	−	+	−	+	+	−
CB9915	CB9915	O123:H11	−	+	+	+	−	−	−	+	−	−	+	−
CB10528	CB10528	O172:H25	−	+	+	−	−	−	−	+	+	−	−	−

### Beef samples, enrichment, and DNA extraction

A set of 1739 beef samples composed of ground beef and carcasses were collected from routine screening using the GeneDisc array (Pall GeneDisc, Bruz, France) at the Veterinary Departmental Laboratory of Touraine, France during a 1-year period as well as in meat production plants. For this study, sampling was biased to get greater numbers of DNA samples positive for *stx* alone (*n* = 306), positive for *stx* and *eae* (*n* = 180), positive for *eae* alone (*n* = 200), and negative for both *stx* and *eae* (*n* = 1053). This sampling scheme does not represent the prevalence of STEC and EHEC in French beef. All samples were incubated in buffered peptone water (BioMerieux, Marcy l'étolie, France) for 18–24 h at 37°C. After enrichment, DNA was extracted from 1 ml of enriched sample using the InstaGene matrix (Bio-Rad Laboratories, Marnes-La Coquette, France) following manufacturer's instruction and DNA was stored at −20°C until use. When samples were found positive for *stx, eae*, and *rfbE*_O157_, isolation of strains was attempted by local laboratories for confirmation of EHEC O157:H7. Following the recommendation of the French ministry of agriculture the appropriate sanitary measures were taken in positive cases of EHEC O157:H7. Unfortunately, because this study was performed several months after the samples were collected, the original samples were not conserved to attempt isolation from presumptive positives with the alternate methods described in this study.

### High-throughput real-time PCR

A LightCycler® 1536 (Roche, Meylan, France) was used to perform high throughput real-time PCR amplifications as described previously (Delannoy et al., 2012a), except that 1 μl of sample DNA was used in each reaction for a final reaction volume of 2 μl. The thermal profile was modified as follows: 95°C for 1 min, followed by 45 cycles of 95°C for 0 s, and 58°C for 30 s. All ramp rates were set to 2°C/s. *E. coli* gene targets used for the real-time PCR amplification and all primers and probes that have previously been described are reported in Table 2. An inhibition control (IC) was performed on each sample to check for potential inhibition of the PCR reaction due to intrinsic characteristics of the sample. The IC is a recombinant pBluescript IISK+ plasmid containing the *dsb* gene from *Ehrlichia canis* (Michelet et al., 2014). The plasmid was added to each sample at a concentration of approximately 0.3 pg/μl. Primers and probe specific for the *E. canis dsb* gene were used to detect the IC (Michelet et al., 2014).

**Table 2:** **Primer and probe sequences used in this study**.

**Primer or probe^a^**	**Sequence 5′ → 3′^b^**
*stx1*_F^c^	TTTGTYACTGTSACAGCWGAAGCYTTACG
*stx1*_R^c^	CCCCAGTTCARWGTRAGRTCMACRTC
*stx1*_P^c^	CTGGATGATCTCAGTGGGCGTTCTTATGTAA
*stx2*_F^c^	TTTGTYACTGTSACAGCWGAAGCYTTACG
*stx2*_R^c^	CCCCAGTTCARWGTRAGRTCMACRTC
*stx2*_P^c^	TCGTCAGGCACTGTCTGAAACTGCTCC
*eae*_F^d^	CATTGATCAGGATTTTTCTGGTGATA
*eae*_R^d^	CTCATGCGGAAATAGCCGTTA
*eae*_P^d^	ATAGTCTCGCCAGTATTCGCCACCAATACC
*eae*-beta_F^d^	GGTGATAATCAGAGTGCGACATACA
*eae*-beta_R^d^	GGCATCAAAATACGTAACTCGAGTAT
*eae*-beta_P^d^	CCACAGCAATTACAATACTACCCGGTGCA
*eae*-gamma_F^d^	GACTGTTAGTGCGACAGTCAGTGA
*eae*-gamma_R^d^	TTGTTGTCAATTTTCAGTTCATCAAA
*eae*-gamma_P^d^	TGACCTCAGTCGCTTTAACCTCAGCC
*eae*-epsilon_F^d^	ATACCCAAATTGTGAAAACGGATA
*eae*-epsilon_R^d^	CACTAACAACAGCATTACCTGCAA
*eae*-epsilon_P^d^	CCAGATGTCAGTTTTACCGTAGCCCTACCA
*eae*-theta_F^d^	TGTTAAAGCACCTGAGGTTACATTTT
*eae*-theta_R^d^	TCACCAGTAACGTTCTTACCAAGAA
*eae*-theta_P^d^	TCAACCTTGTTGTCAATTTTCAGTCCATCA
*espK*_F^e^	GCAGRCATCAAAAGCGAAATCACACC
*espK*_R^e^	TCGTTTGGTAACTGTGGCAGATACTC
*espK*_P^e^	ATTCAGATAGAAGAAGCGCGGGCCAG
*espV*_F^e^	TCAGGTTCCTCGTCTGATGCCGC
*espV*_R^e^	CTGGTTCAGGCCTGGAGCAGTCC
*espV*_P^e^	CTTGCAACACGTTACGCTGCCGAGTATT
*Z2098*_F^f^	CTGAAAAGAGCCAGAACGTGC
*Z2098*_R^f^	TGCCTAAGATCATTACCCGGAC
*Z2098*_P^f^	TAACTGCTATACCTCCGCGCCG
*ureD*_F^e^	GCAATAATTGACTCTGATTGCC
*ureD*_R^e^	GCTGCTGCGGTAAAATTTACT
*ureD*_P^e^	TACGCTGATCACCATGCCTGGTGC
SP_O26-E_F^g^	AAACCGATCTCCTCATCCTC
SP_O26-E_R^h^	ATCAACATGCAGCGCGAACG
SP_O26-E_P^g^	CCAGCTACCGACAGTAGTGTGTTCC
rfbE_*O*157_-F^c^	TTTCACACTTATTGGATGGTCTCAA
rfbE_*O*157_-R^c^	CGATGAGTTTATCTGCAAGGTGAT
rfbE_*O*157_-P^c^	AGGACCGCAGAGGAAAGAGAGGAATTAAGG
wzx_*O*26_-F^c^	CGCGACGGCAGAGAAAATT
wzx_*O*26_-R^c^	AGCAGGCTTTTATATTCTCCAACTTT
wzx_*O*26_-P^c^	CCCCGTTAAATCAATACTATTTCACGAGGTTGA
wzx_*O*103_-F^i^	CAAGGTGATTACGAAAATGCATGT
wzx_*O*103_-R^i^	GAAAAAAGCACCCCCGTACTTAT
wzx_*O*103_-P_*i*_	CATAGCCTGTTGTTTTAT
wbdl_*O*111_-F^c^	CGAGGCAACACATTATATAGTGCTTT
wbdl_*O*111_-R^c^	TTTTTGAATAGTTATGAACATCTTGTTTAGC
wbdl_*O*111_-P^c^	TTGAATCTCCCAGATGATCAACATCGTGAA
wzx_*O*121_-F^j^	TGGTCTCTTAGACTTAGGGC
wzx_*O*121_-R^j^	TTAGCAATTTTCTGTAGTCCAGC
wzx_*O*121_-P^j^	TCCAACAATTGGTCGTGAAACAGCTCG
wzx_*O*45_-F^j^	TACGTCTGGCTGCAGGG
wzx_*O*45_-R^j^	ACTTGCAGCAAAAAATCCCC
wzx_*O*45_-P^j^	TTCGTTGCGTTGTGCATGGTGGC
wzy_*O*145_-F^k^	ATATTGGGCTGCCACTGATGGGAT
wzy_*O*145_-R^k^	TATGGCGTACAATGCACCGCAAAC
wzy_*O*145_-P^k^	AGCAGTGGTTCGCGCACAGCATGGT

a F, forward primer; R, reverse primer; P, probe.

b All probes were labeled with 6-HEX or 6-FAM and BHQ1 (Black Hole Quencher).

c Oligonucleotide described by Perelle et al. (2004).

d Oligonucleotide described by Nielsen and Andersen (2003).

e Oligonucleotide described by Delannoy et al. (2013a).

f Oligonucleotide described by Delannoy et al. (2013b).

g Oligonucleotide described by Delannoy et al. (2012a).

h Oligonucleotide described by Delannoy et al. (2015a).

i Oligonucleotide described by Perelle et al. (2005).

j Oligonucleotide described by Bugarel et al. (2010).

k Oligonucleotide described by Fratamico et al. (2009).

## Results

### Presence of *stx1, stx2, eae, espK, espV, Z2098*, and *ureD* in *E. coli* strains

The presence of *stx1, stx2, eae, espK, espV, Z2098*, and *ureD* was tested by PCR in a panel of *E. coli* strains obtained from culture collections (Table 1). All *E. coli* strains used in this study were positive for the *stx* and *eae* genes and therefore can be considered as typical EHEC strains. Strains were associated with the following *eae* variants: *eae*-beta (O26:H11, O26:H_ND_, O103:H11, O5, O118:H16, O123:H11), *eae*-gamma (O145:H28, O145:H_ND_, O157:H7, O55:H7), *eae*-epsilon (O121:H19, O45:H2, O103:H2, O103:H11), and *eae*-theta (O111:H8, O111:H_ND_, O103:H25). The *eae* subtype of strain CB10528 (O172:H25) could not be determined. Distribution of the genetic markers *espK, espV, Z2098*, and *ureD* in the 55 EHEC strains is shown in Table 1. Overall, the genetic markers investigated were detected in most of the EHEC strains examined. With the exception of the new O26:H11 *stx2*-positive strain (CB14699) and one strain of serotype O157:H7 (CDC-C984), all of the strains were positive for *espK*. The *espV* gene was not detected in two *E. coli* O26:H11 strains (ATCC2196 and CB14699), in O45:H2, O118:H16, O5, or in O123:H11 isolates. The *Z2098* gene marker tested negative in only a few strains: the new O26:H11 *stx2* positive clone (strain CB14699) and in the O55:H7 strains. The *ureD* gene was absent in the new O26:H11 *stx2* positive clone (strain CB14699) and in the O55:H7 strains. It was also absent from a few O103:H2 isolates (strains PMK5, CB12062, CB12092). In summary, all of the strains were positive for one or more of the genes *espK, espV, Z2098*, and *ureD*, with the exception of the new O26:H11 *stx2* positive clone (CB14699).

### Screening beef enrichments for *stx1, stx2, eae, espK, espV, Z2098*, and *ureD*

**Figure 1:**
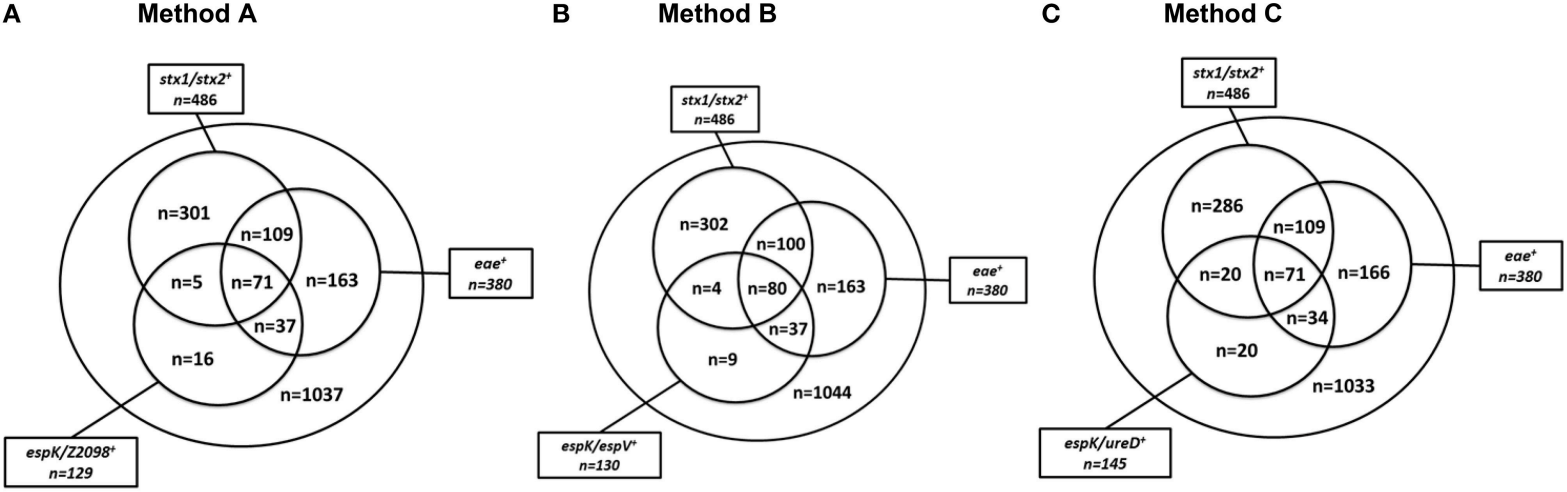
**Distribution of the genetic markers *stx*, *eae*, *espK*, *espV*, and *Z2098* among 1739 beef samples. (A)** (*stx1*/*stx2, eae*, and *espK*/*Z2098*) is Method A, **(B)** (*stx1*/*stx2, eae*, and *espK*/*espV*) is Method B, **(C)** (*stx1*/*stx2, eae*, and *espK*/*ureD*) is Method C. *stx*1/*stx*2^+^ for samples giving a positive result for *stx1* and/or *stx2, eae*^+^ for samples giving a positive result for *eae, espK*/*Z2098*^+^ for samples giving a positive result for *espK* and/or *Z2098, espK*/*espV*^+^ for samples giving a positive result for *espK* and/or *espV, espK*/*ureD*^+^ for samples giving a positive result for *espK* and/or *ureD*.

A set of 1739 beef samples was screened for the presence of *stx1, stx2, eae, espK, espV, Z2098*, and *ureD*. The *stx* genes were detected in 27.95% of the samples (486/1739). The *eae* gene was detected in 21.85% of the samples (380/1739). The two genes were simultaneously present in 10.35% of the samples (180/1739). The genes *espK* and/or *Z2098* were detected in 7.42% of the samples (129/1739) (Figure 1A), while *espK* and/or *espV* were found in 130 samples (7.48%) (Figure 1B) and *espK* and/or *ureD* was recorded in 145 samples (8.34%) (Figure 1C).

By using the *stx* and *eae* genes for screening beef samples, following the ISO/CEN TS13136 and MLG5B.05 methods, 180 samples (10.35%) were recorded as *stx*/*eae* positive and should, therefore, be subjected to a second screening step for EHEC-serogroups. Pre-screening of *stx*/*eae* positive samples for *espK* with either *Z2098* (alternate method A), or *ureD* (alternate method C), provided a 60% reduction of the number of samples that should be submitted to a second screening targeting the O-group gene markers (*n* = 71). Using the alternate method B (*stx*/*eae*/*espK*/*espV*), 80 of the 1739 samples (4.6%) needed to be submitted to a further screening for serogroup determination, which represents a reduction by 55% of the number of samples subjected to a second screening.

**Figure 2:**
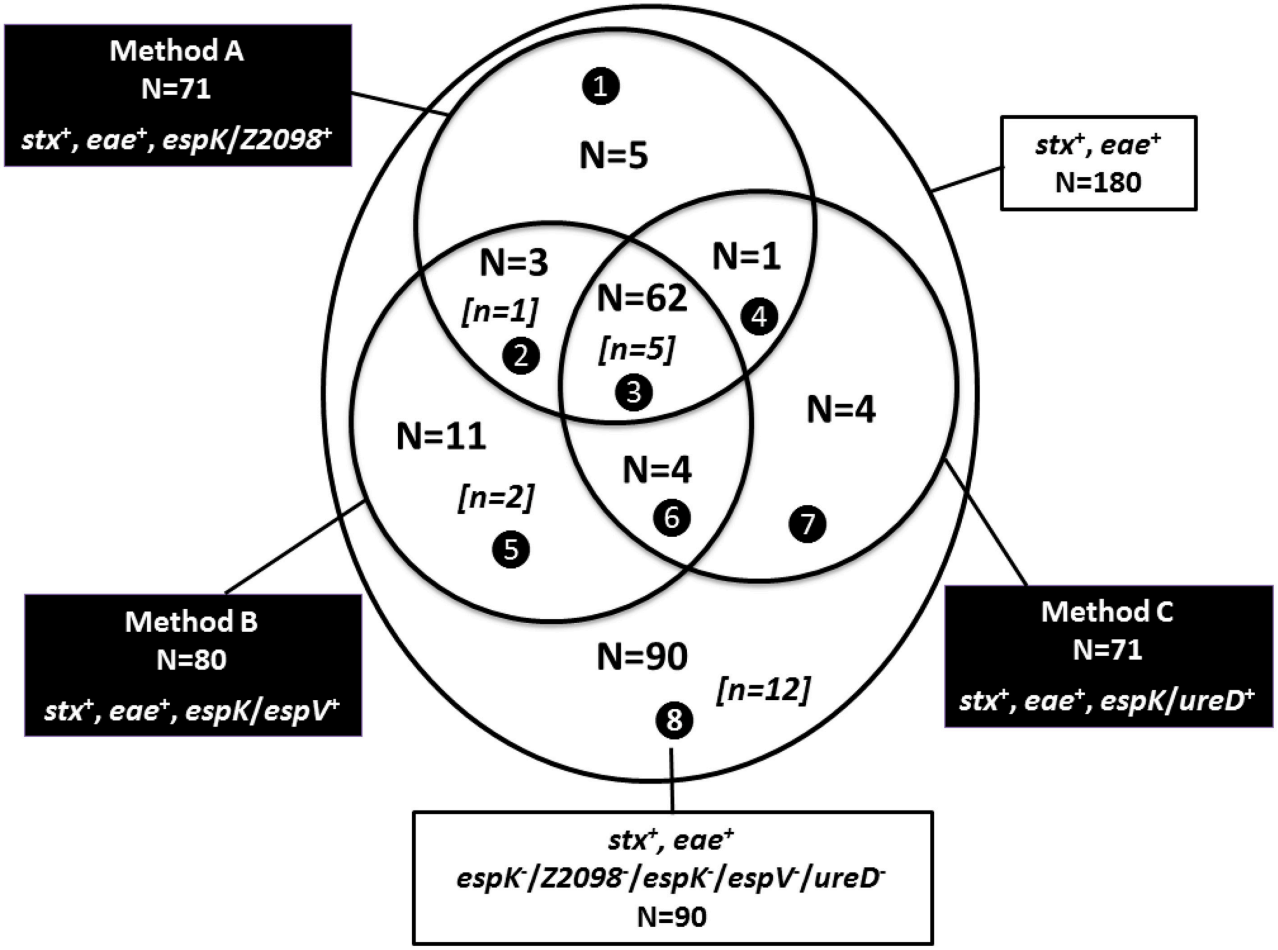
**Comparison of Methods A–C on 180 beef samples that tested positive for both *stx* and *eae* genes**. *stx*^+^ for samples giving a positive result for *stx1* and/or *stx2*; *eae*^+^ for samples giving a positive result for *eae*; *espK*/*Z2098*^+^ for samples giving a positive result for *espK* and/or *Z2098*; *espK*/*espV*^+^ for samples giving a positive result for *espK* and/or *espV*; *espK*/*ureD*^+^ for samples giving a positive result for *espK* and/or *ureD*. Sector ❶ (*stx*^+^, *eae*^+^, *Z2098*^+^), sector ❷ (*stx*^+^, *eae*^+^, *Z2098*^+^, *espV*^+^), sector ❸ (*stx*^+^, *eae*^+^, *espK*/*Z2098*^+^, *espK*/*espV*^+^, *espK*/*ureD*^+^), sector ❹ (*stx*^+^, *eae*^+^, *Z2098*^+^, *ureD*^+^), sector ❺ (*stx*^+^, *eae*^+^, *espV*^+^), sector ❻ (*stx*^+^, *eae*^+^, *espV*^+^, *ureD*^+^), sector ❼ (*stx*^+^, *eae*^+^, *ureD*^+^) and sector ❽ (*stx*^+^, *eae*^+^). *[n* = *x]* is the number of samples that tested positive by the CRISPR_O26:H11_ PCR assay detecting the new O26 clone, *N* = *x* is the total number of samples per sector.

Figure 2 shows the comparison of the alternative methods A–C with the 180 beef samples that tested positive for both the *stx* and *eae* genes. A total of 90 *stx* and *eae* positive beef samples tested negative for *esp*K, *esp*V, *Z2098*, and *ure*D (sector 8, Figure 2). However, the inclusion of the CRISPR_O26:H11_ PCR revealed 12 *esp*K, *esp*V, *Z2098*, and *ure*D negative samples that were positive for both the new CRISPR_O26:H11_ clone and *eae*-beta, the variant of the intimin gene carried by EHEC O26:H11 (see below).

### Screening beef samples for *stx1, stx2, eae*, and CRISPR_O26:H11_

The 180 beef samples that are positive for *stx* and *eae* were also tested by the CRISPR_O26:H11_ PCR test (SP_O26-E, as described in Delannoy et al., 2015a), that detects the new EHEC O26:H11 French clone (*stx2* and *eae* positive, *espK, espV, Z2098, ureD* negative). Among the 180 samples tested, 20 *stx*/*eae* positive samples were also found to be positive for SP_O26-E and should therefore be submitted to a further screening for serogroup determination. Interestingly, most of them (16/20) were found positive for *stx2*, 2 samples were positive for stx1 only and 2 had an unknown *stx* subtype. Twelve of these 20 were also negative for *espK, espV, Z2098*, and *ureD* (Figure 2, sector 8). Finally, when combining the use of CRISPR_O26:H11_ (SP_O26-E PCR test) with alternate method A, 85 samples should be submitted to a second screening targeting the O-group gene markers. When using alternate methods B and C with the CRISPR_O26:H11_ PCR test, 92 and 86 samples should be submitted to the second step, respectively.

### Screening beef samples for *stx1, stx2, eae*, and the top seven EHEC serogroups

As recommended in the ISO/TS 13136 (EU) and MLG5B.05 (US) reference methods, the 180 *stx* and *eae* positive samples were tested for the top seven EHEC serogroups. Among these, 115 samples were positive for at least one of the top seven US regulated EHEC serogroups and 99 were positive for at least one of the top five EHEC serogroups screened by the European ISO/TS 13136 method (data not shown). The most frequently found serogroup was O103 (*n* = 71), followed by O26 (*n* = 45), O121 (*n* = 38), O157 (*n* = 18), O45 (*n* = 14), O145 (*n* = 6), and O111 (*n* = 1). Interestingly, 30 samples were positive for 2 serogroups, 11 were positive for 3 serogroups, and 7 for more than 4 serogroups. In final, 41.74% (48/115) of the beef samples tested positive for more than one O-group marker.

### Screening beef samples for *stx1, stx2, eae*, and the *eae* subtypes

The 180 *stx* and *eae* positive beef samples were further tested for the *eae* subtypes gamma, beta, epsilon and theta, which are associated with one or more of the top seven EHEC serogroups. Among these 180 *stx* and *eae* positive samples, 135 tested positive for at least one of the four *eae*-subtypes: gamma, beta, epsilon and theta (data not shown). The most frequently detected *eae*-subtypes were *eae*-beta (*n* = 94) and *eae*-theta (*n* = 65), followed by *eae*-epsilon (*n* = 15), and *eae*-gamma (*n* = 5). Trying to correlate the *eae* subtype with the serogroup, we identified 51 beef samples for which at least one serogroup was associated with the corresponding related *eae*-subtype. Among these 51 samples, 6 were positive with multiple serogroups.

### Comparison of alternative methods A–C for screening beef samples

We identified 62 samples that were recorded positive with the three alternative methods A–C (sector 3, Figure 2) and therefore must be submitted to a second screening targeting the O-group gene markers. These samples are strongly suspected to contain typical EHEC and five of these are also suspected to contain the new CRISPR_O26:H11_ clone. In addition we found 28 samples (sectors 1, 2, 4, 5, 6, 7 from Figure 2) that were positive by one or two alternative methods only. PCR results obtained for these 28 samples for *eae* subtypes and top seven O-groups were as follows: only one sample among the 5 samples of sector 1 (Figure 2) was positive for the association of O26 and *eae*-beta, but it tested negative for the different CRISPR_O26:H11_ assays targeting EHEC O26:H11 [SP_O26-C, SP_O26-D, and SP_O26-E, as described in Delannoy et al., 2012a, 2015a (data not shown)]. The only sample from sector 4 (Figure 2) and each of the four samples from sectors 6 and 7 (Figure 2) were found negative by the association of the top seven serogroups and the corresponding *eae*-subtypes. From sector 2 (Figure 2), only one out of three samples was found positive for the new CRISPR_O26:H11_ clone (this sample tested positive by PCR for O26, *eae*-beta, SP_O26-E; and was also *stx2* positive which is consistent with the new clone). The two other samples were not suspected as “presumptive positive” based on the *eae*-subtypes and top seven EHEC serotypes determination. In sector 5 (Figure 2), out of eleven samples three were found “presumptive positive” for EHEC O26 (among them two were suspected to be positive for the new O26:H11 clone). Finally, “presumptive positive” samples were recorded in sectors 2, 3, and 5 which lead to consider the alternate method B as the best one among the other alternate methods for screening EHEC strains. In order to complete the screening of EHEC and not to exclude the new virulent O26 clone, the alternate method B should include the screening of the new CRISPR_O26:H11_ assay.

## Discussion

A STEC seropathotype classification has been based upon the serotype association with human epidemics, bloody diarrhea, and HUS, and has been developed as a tool to assess the clinical and public health risks associated with non-O157 EHEC and STEC strains (Karmali et al., 2003). This approach has been of considerable value in defining pathogenic STEC serotypes of importance in cases of human infection (EFSA, 2007; Coombes et al., 2011); however it does not resolve the underlying problem with strains that have not yet been fully serotyped. Furthermore, classification based solely on the presence of seropathotype is inadequate with illnesses linked to STEC serotypes other than O157:H7 that are on the rise worldwide, indicating that some of these organisms may be emerging pathogens. In 2013, the Panel on Biological Hazards (BIOHAZ) of the European Food Safety Authority (EFSA) published a Scientific Opinion on “VTEC-seropathotype and scientific criteria regarding pathogenicity assessment” (EFSA, 2013). This document has focused attention on the applicability of the Karmali seropathotype concept. The document does not provide a scientific definition of a pathogenic STEC but states that the seropathotype classification of Karmali et al. (2003) does not define pathogenic STEC nor does it provide an exhaustive list of pathogenic serotypes. It is not possible to fully define human pathogenic STEC or identify factors for STEC that absolutely predict the potential to cause human disease, but strains positive for Shiga-toxin (in particular the *stx2* genes) and *eae* (intimin production) genes are associated with a higher risk of more severe illness than other virulence factor combinations (EFSA, 2013). Severe disease, and particularly HUS, is linked to certain serotypes and strains and this link must be the result of particular genetic factors or combinations of factors that have to be determined. A new molecular classification scheme has been proposed by the EFSA Panel on Biological Hazards (BIOHAZ) that relies more on virulence factors than seropathotypes. It is proposed that STEC serogroups O157, O26, O103, O145, O111, and O104 in combination with *stx* and *eae* or *stx* and both *aaiC* (secreted protein of EAEC) and *aggR* (plasmid-encoded regulator) genes should be considered as presenting a potentially higher risk for bloody diarrhea and HUS, such strains are categorized in group I (EFSA, 2013). For any other serogroups in combination with the same genes, the potential risk is regarded as high for diarrhea, but currently unknown for HUS, such strains are categorized in group II. The inclusion of *aaiC* and *aggR* genes in the proposed molecular approach is due to the O104:H4 outbreak, which was caused by a highly virulent strain (Frank et al., 2011). This appears to be an exceptional event (Prager et al., 2014) and future surveillance will provide data that may be used to review the inclusion of these virulence factors but a recent study showed that French cattle are not a reservoir of the highly virulent enteroaggregative Shiga toxin-producing *E. coli* of serotype O104:H4 (Auvray et al., 2012).

Following the EFSA opinion, several laboratories have attempted to develop detection and identification methods for strains of groups I and II, and although substantial progress has been made, a practical method of pathogenic STEC detection has yet to be validated. Molecular methods for screening EHEC O157 and non-O157 in beef products rely currently on the molecular detection of *stx, eae*, and the top five or top seven EHEC serogroups in mixed bacterial enrichments as described in the ISO/TS 13136 (EU) and MLG5B.05 (US) reference methods (ISO, 2012; USDA-FSIS, 2014), followed by attempted isolation of the correct strain. These approaches bear the disadvantage that food samples that carry mixtures of *stx*-negative *E. coli* carrying *eae* genes together with *eae*-negative STEC falsely indicate the presence of EHEC when analyzed for the genes mentioned above (Beutin et al., 2009; Fratamico and Bagi, 2012; Wasilenko et al., 2014). Hence, screening food enrichment broths for *stx* and *eae* genes may cause needless disruption and costs for food producers when the risk of low-virulence STEC is overestimated. The erroneous identification of cucumbers as the source in the outbreak in Germany in 2011 cost European fruit and vegetable producers approximately € 812 million (Commission of the European Communities., 2011). Thus, new DNA targets that unambiguously identify typical EHEC strains (*stx*-positive and *eae*-positive *E. coli* strains) in complex samples are a desirable goal. This new panel of genes might include novel genetic markers recently identified as highly associated to pathogenic STEC strains (Delannoy et al., 2013a,b). Thus, the associations of *espK* with either *espV, ureD*, or *Z2098* were found to be the best combinations for more specific and sensitive detection of the top seven EHEC strains, allowing detection of 99.3–100% of these strains. In addition, detection of 93.7% of the typical EHEC strains belonging to other serotypes than the top seven offered the possibility for identifying new emerging typical EHEC strains (Delannoy et al., 2013a,b). Conversely, these different combinations of genetic markers were very rarely associated with STEC (1.6–3.6%) and with non-pathogenic *E. coli* (1.1–3.4%). The objective of the present study was to refine the EHEC screening systems for testing beef samples via the incorporation of these additional gene targets *espK, espV, ureD*, and *Z2098* in the detection scheme. In addition to these four targets, we included a CRISPR_O26:H11_ target in the detection scheme that has been designed for detecting a new clone of STEC O26:H11 harboring *stx2* only and strongly associated with HUS in France (Delannoy et al., 2015a,b). This new EHEC O26:H11 French clone is positive for *eae* (*eae*-beta subtype) but does not contain any of the above markers *espK, espV, ureD*, or *Z2098*. The fact that the new French clone is missing *espK, espV, ureD*, and *Z2098* does not mean that these genes are not required EHEC virulence factors. We have previously demonstrated that they are significantly associated with EHEC strains and not the other pathotypes. It does suggest however that the new French clone may harbor a different set of virulence genes (just like atypical EHEC lack *eae*), which can be investigated through additional genomic studies. In the meantime, although CRISPR sequences are not virulence factors *per se*, we have demonstrated that certain specific spacers are associated with EHEC strains from the top7 serotypes and thus can provide specific and sensitive detection of the top 7 EHEC strains (Delannoy et al., 2012a,b).

To validate the pertinence of this new approach we screened 1739 beef samples and collected 180 samples that tested positive for both *stx* and *eae*, and must be subjected to a second screening step for serogroup determination according to the ISO/TS 13136 (EU) and MLG5B.05 (US) reference methods (ISO, 2012; USDA-FSIS, 2014). Among these 180 samples, 135 tested positive for at least one of the four *eae*-subtypes gamma, beta, epsilon and theta, which are related to typical EHEC and in particular to those of the top seven serogroups (Bugarel et al., 2010). Introduction of the *eae*-subtypes in the screening step provided a reduction by 25% of the number of *stx*/*eae* positive samples that should be subjected to a further screening for serogroup determination. A more significant refinement of the first EHEC screening step was achieved by including *espK, espV, ureD, Z2098*, and CRISPR_O26:H11_ target genes in the detection scheme. Thus, a reduction by 52.78–52.22% of the number of samples subjected to a further screening for serogroup determination was obtained by using respectively the alternate method A (*stx*/*eae*/*espK*/*Z2098*) or method C (*stx*/*eae*/*espK*/*ureD*) in combination with the CRISPR_O26:H11_ PCR assay detecting the new O26 clone. A reduction by 48.88% of the number of “presumptive positive” samples was obtained using the alternate method B (*stx*/*eae*/*espK*/*espV*) in association with the CRISPR_O26:H11_ PCR assay. Given the additional information on the association of the top seven serogroups and the *eae*-subtypes, we determined the last approach, i.e., method B (*stx*/*eae*/*espK*/*espV*) in association with the CRISPR_O26:H11_ PCR assay, as the best approach to narrow down the EHEC screening step in beef samples. Using such an approach, 92 samples must be subjected to a further screening for serogroup determination vs. 180 with the conventional *stx*/*eae* approach used in the ISO/TS 13136 (EU) and MLG5B.05 (US) reference methods (ISO, 2012; USDA-FSIS, 2014). This constitutes a significant reduction (almost 50%) of the number of samples subjected to a second screening targeting the O-group gene markers. Moreover, this approach is in line with the EFSA opinion that has identified STEC strains of groups I and II as presenting the potential higher risk for diarrhea and HUS (EFSA, 2013). Identification of additional gene markers, i.e., *espK, espV*, and CRISPR_O26:H11_ to better distinguish typical EHEC from other *E. coli* pathogroups would substantially enhance the power of EHEC test systems providing a significant reduction of “presumptive positive” in beef samples. Such a new approach would provide to the agroindustry a novel method for tracking EHEC in food samples. This work should be considered with interest to draw up the outline of a future standard that will follow the recommendations of EFSA.

## Author contributions

Conceived and designed the experiments: SD, PF. Performed the experiments: SD, BC, SI, HW, JD, IB. Analyzed the data: SD, BC, PF. Contributed reagents/materials/analysis tools: SD, LB, JD, IB, PF. Wrote the paper: SD, PF. Critical revision of the paper for important intellectual content: SD, BC, SI, HW, LB, JD, IB, PF.

## Funding

The project was partially financed by the French “joint ministerial program of R&D against CBRNE risks” (Grant number C17609-2).

### Conflict of interest statement

The authors declare that the research was conducted in the absence of any commercial or financial relationships that could be construed as a potential conflict of interest.

## References

[B1] Auvray F. Dilasser F. Bibbal D. Kérourédan M. Oswald E. Brugère H. (2012). French cattle is not a reservoir of the highly virulent enteroaggregative Shiga toxin-producing *Escherichia coli* of serotype O104:H4. Vet Microbiol. 158, 443–445. 10.1016/j.vetmic.2012.02.029 22424867

[B2] Beutin L, Fach, P. (2014). Detection of shiga toxin-producing *Escherichia coli* from nonhuman sources and strain typing. Microbiol. Spectr. 2:EHEC-0001-2013. 10.1128/microbiolspec.EHEC-0001-2013 26103970

[B3] Beutin L. Jahn S. Fach P. (2009). Evaluation of the ‘GeneDisc’ real-time PCR system for detection of enterohaemorrhagic *Escherichia coli* (EHEC) O26, O103, O111, O145 and O157 strains according to their virulence markers and their O- and H-antigen-associated genes. J. Appl. Microbiol. 106, 1122–1132. 10.1111/j.1365-2672.2008.04076.x 19191965

[B4] Bibbal D. Loukiadis E. Kérourédan M. Peytavin de Garam C. Ferré F. Cartier P.. (2014). Intimin gene (*eae*) subtype-based real-time PCR strategy for specific detection of Shiga toxin-producing *Escherichia coli* serotypes O157:H7, O26:H11, O103:H2, O111:H8, and O145:H28 in cattle feces. Appl. Environ. Microbiol. 80, 1177–1184. 10.1128/AEM.03161-13 24296503 PMC3911233

[B5] Bielaszewska M. Mellmann A. Bletz S. Zhang W. Köck R. Kossow A.. (2013). Enterohemorrhagic *Escherichia coli* O26:H11/H-: a new virulent clone emerges in Europe. Clin. Infect. Dis. 56, 1373–1381. 10.1093/cid/cit055 23378282

[B6] Brooks J. T. Sowers E. G. Wells J. G. Greene K. D. Griffin P. M. Hoekstra R. M.. (2005). Non-O157 Shiga toxin-producing *Escherichia coli* infections in the United States, 1983-2002. J. Infect. Dis. 192, 1422–1429. 10.1086/466536 16170761

[B7] Bugarel M. Beutin L. Fach P. (2010). Low-density macroarray targeting non-locus of enterocyte effacement effectors (nle genes) and major virulence factors of Shiga toxin-producing *Escherichia coli* (STEC): a new approach for molecular risk assessment of STEC isolates. Appl. Environ. Microbiol. 76, 203–211. 10.1128/AEM.01921-09 19880649 PMC2798666

[B8] Commission of the European Communities (2011). Commission Staff Working Document on Lessons Learned from the 2011 Outbreak of Shiga Toxin-Producing Escherichia coli (STEC) O104:H4 in Sprouted Seeds. SANCO/13004/2011, 1–23. Available online at http://ec.europa.eu/food/food/biosafety/salmonella/docs/cswd_lessons_learned_en.pdf (Accessed on August 8, 2015).

[B9] Coombes B. K. Gilmour M. W. Goodman C. D. (2011). The evolution of virulence in non-o157 shiga toxin-producing *Escherichia coli*. Front. Microbiol. 2:90. 10.3389/fmicb.2011.00090 21833329 PMC3153049

[B10] Delannoy S. Beutin L. Burgos Y. Fach P. (2012b). Specific detection of enteroaggregative hemorrhagic *Escherichia coli* O104:H4 strains by use of the CRISPR locus as a target for a diagnostic real-time PCR. J. Clin. Microbiol. 50, 3485–3492. 10.1128/JCM.01656-12 22895033 PMC3486251

[B11] Delannoy S. Beutin L. Fach P. (2012a). Use of clustered regularly interspaced short palindromic repeat sequence polymorphisms for specific detection of enterohemorrhagic *Escherichia coli* strains of serotypes O26:H11, O45:H2, O103:H2, O111:H8, O121:H19, O145:H28, and O157:H7 by real-time PCR. J. Clin. Microbiol. 50, 4035–4040. 10.1128/JCM.02097-12 23035199 PMC3503007

[B12] Delannoy S. Beutin L. Fach P. (2013a). Discrimination of enterohemorrhagic *Escherichia coli* (EHEC) from non-EHEC strains based on detection of various combinations of type III effector genes. J. Clin. Microbiol. 51, 3257–3262. 10.1128/JCM.01471-13 23884997 PMC3811616

[B13] Delannoy S. Beutin L. Fach P. (2013b). Towards a molecular definition of enterohemorrhagic *Escherichia coli* (EHEC): detection of genes located on O island 57 as markers to distinguish EHEC from closely related enteropathogenic *E. coli* strains. J. Clin. Microbiol. 51, 1083–1088. 10.1128/JCM.02864-12 23325824 PMC3666763

[B14] Delannoy S. Mariani-Kurkdjian P. Bonacorsi S. Liguori S. Fach P. (2015a). Characteristics of emerging human-pathogenic *Escherichia coli* O26:H11 strains isolated in France between 2010 and 2013 and carrying the stx2d gene only. J. Clin. Microbiol. 53, 486–492. 10.1128/JCM.02290-14 25428148 PMC4298503

[B15] Delannoy S. Mariani-Kurkdjian P. Bonacorsi S. Liguori S. Ison S. A. Fach P. (2015b). Draft genome sequences of human-pathogenic *Escherichia coli* O26:H11 strains carrying the stx2 gene only and circulating in France. Genome Announc. 3:e00852-15. 10.1128/genomeA.00852-15 26227606 PMC4520904

[B16] EFSA (European Food Safety Authority) (2007). Scientific Opinion of the Panel on Biological Hazards (BIOHAZ) on monitoring of verotoxigenic *Escherichia coli* (VTEC) and identification of human pathogenic VTEC types. EFSA J. 579, 1–61. 10.2903/j.efsa.2007.579

[B17] EFSA (European Food Safety Authority) (2013). Scientific Opinion of the Panel on Biological Hazards (BIOHAZ) on VTEC-seropathotype and scientific criteria regarding pathogenicity assessment. EFSA J. 11, 3138, 1–106. 10.2903/j.efsa.2013.3138

[B18] EFSA (European Food Safety Authority European Centre for Disease Prevention Control). (2014). The European Union Summary Report on Trends and Sources of Zoonoses, Zoonotic Agents and Food-borne Outbreaks in 2012. EFSA J. 12, 3547, 312. 10.2903/j.efsa.2014.3547 PMC7009540 32625785

[B19] Frank C. Werber D. Cramer J. P. Askar M. Faber M. an der Heiden M.. (2011). Epidemic profile of Shiga-toxin-producing *Escherichia coli* O104:H4 outbreak in Germany. N. Engl. J. Med. 365, 1771–1780. 10.1056/NEJMoa1106483 21696328

[B20] Fratamico P. M. Bagi L. K. (2012). Detection of Shiga toxin-producing *Escherichia coli* in ground beef using the GeneDisc real-time PCR system. Front. Cell Infect. Microbiol. 2:152. 10.3389/fcimb.2012.00152 23267438 PMC3526733

[B21] Fratamico P. M. DebRoy C. Miyamoto T. Liu Y. (2009). PCR detection of enterohemorrhagic *Escherichia coli* O145 in food by targeting genes in the *E. coli* O145 O-antigen gene cluster and the shiga toxin 1 and shiga toxin 2 genes. Foodborne Pathog Dis. 6, 605–611. 10.1089/fpd.2008.0254 19435408

[B22] Gould L. H. Mody R. K. Ong K. L. Clogher P. Cronquist A. B. Garman K. N.. (2013). Increased recognition of non-O157 Shiga toxin-producing *Escherichia coli* infections in the United States during 2000–2010: epidemiologic features and comparison with *E. coli* O157 infections. Foodborne Pathog. Dis. 10, 453–460. 10.1089/fpd.2012.1401 23560425

[B23] ISO (International Organization for Standardization) (2012). ISO/TS 13136:2012, Microbiology of Food and Animal Feed—Real-Time Polymerase Chain Reaction (PCR) –Based Method for the Detection of Food-Borne Pathogens—Horizontal Method for the Detection of Shiga Toxin-Producing Escherichia coli (STEC) and the Determination of O157, O111, O26, O103, and O145 Serogroups. Available online at: http://www.iso.org/iso/iso_catalogue/catalogue_tc/catalogue_detail.htm?cs number=53328. (Accessed on August 8, 2015).

[B24] Johnson K. E. Thorpe C. M. Sears C. L. (2006). The emerging clinical importance of non-O157 Shiga toxin-producing *Escherichia coli*. Clin. Infect. Dis. 43, 1587–1595. 10.1086/509573 17109294

[B25] Karmali M. A. Mascarenhas M. Shen S. Ziebell K. Johnson S. Reid-Smith R.. (2003). Association of genomic O island 122 of *Escherichia coli* EDL 933 with verocytotoxin-producing *Escherichia coli* seropathotypes that are linked to epidemic and/or serious disease. J. Clin. Microbiol. 41, 4930–4940. 10.1128/JCM.41.11.4930-4940.2003 14605120 PMC262514

[B26] Livezey K. W, Groschel, B. Becker M. M. (2015). Use of the ecf1 gene to detect Shiga toxin-producing *Escherichia coli* in beef samples. J. Food Prot. 78, 675–684. 10.4315/0362-028X.JFP-14-417 25836391

[B27] Madic J. Peytavin de Garam C. Vingadassalon N. Oswald E. Fach P. Jamet E.. (2010). Simplex and multiplex real-time PCR assays for the detection of flagellar (H-antigen) fliC alleles and intimin (*eae*) variants associated with enterohaemorrhagic *Escherichia coli* (EHEC) serotypes O26:H11, O103:H2, O111:H8, O145:H28 and O157:H7. J. Appl. Microbiol. 109, 1696–1705. 10.1111/j.1365-2672.2010.04798.x 20618885

[B28] Madic J. Vingadassalon N. Peytavin de Garam C. P. Marault M. Scheutz F. Brugère H.. (2011). Detection of Shiga toxin-producing *Escherichia coli* serotypes O26:H11, O103:H2, O111:H8, O145:H28, and O157:H7 in raw-milk cheeses by using multiplex real-time PCR. Appl. Environ. Microbiol. 77, 2035–2041. 10.1128/AEM.02089-10 21239543 PMC3067316

[B29] Michelet L. Delannoy S. Devillers E. Umhang G. Aspan A. Juremalm M.. (2014). High-throughput screening of tick-borne pathogens in Europe. Front. Cell Infect. Microbiol. 4:103. 10.3389/fcimb.2014.00103 25120960 PMC4114295

[B30] Miko A. Delannoy S. Fach P. Strockbine N. A. Lindstedt B. A. Mariani-Kurkdjian P.. (2013). Genotypes and virulence characteristics of Shiga toxin-producing *Escherichia coli* O104 strains from different origins and sources. Int. J. Med. Microbiol. 303, 410–421. 10.1016/j.ijmm.2013.05.006 23777812

[B31] Nielsen E. M. Andersen M. T. (2003). Detection and characterization of verocytotoxin-producing *Escherichia coli* by automated 5′ nuclease PCR assay. J. Clin. Microbiol. 41, 2884–2893. 10.1128/JCM.41.7.2884-2893.2003 12843017 PMC165313

[B32] Oswald E. Schmidt H. Morabito S. Karch H. Marchès O. Caprioli A. (2000). Typing of intimin genes in human and animal enterohemorrhagic and enteropathogenic *Escherichia coli*: characterization of a new intimin variant. Infect. Immun. 68, 64–71. 10.1128/IAI.68.1.64-71.2000 10603369 PMC97102

[B33] Perelle S. Dilasser F. Grout J. Fach P. (2004). Detection by 5′-nuclease PCR of Shiga-toxin producing *Escherichia coli* O26, O55, O91, O103, O111, O113, O145 and O157:H7, associated with the world's most frequent clinical cases. Mol. Cell Probes. 18, 185–192. 10.1016/j.mcp.2003.12.004 15135453

[B34] Perelle S. Dilasser F. Grout J. Fach P. (2005). Detection of *Escherichia coli* serogroup O103 by real-time polymerase chain reaction. J. Appl. Microbiol. 98, 1162–1168. 10.1111/j.1365-2672.2005.02545.x 15836486

[B35] Prager R. Lang C. Aurass P. Fruth A. Tietze E. Flieger A. (2014). Two novel EHEC/EAEC hybrid strains isolated from human infections. PLoS ONE 9:e95379. 10.1371/journal.pone.0095379 24752200 PMC3994036

[B36] USDA-FSIS (2014). Detection and Isolation of non-O157 Shiga Toxin-Producing Escherichia coli (STEC) from Meat Products and Carcass and Environmental Sponges. In Microbiological Laboratory Guidelines chapter 5B.05. Available online at http://www.fsis.usda.gov/wps/wcm/connect/7ffc02b5-3d33-4a79-b50c-81f208893204/MLG-5B.pdf?MOD=AJPERES (Accessed on December 10, 15).

[B37] Wang F. Yang Q. Kase J. A. Meng J. Clotilde L. M. Lin A.. (2013). Current trends in detecting non-O157 Shiga toxin-producing *Escherichia coli* in food. Foodborne Pathog. Dis. 10, 665–677. 10.1089/fpd.2012.1448 23755895

[B38] Wasilenko J. L. Fratamico P. M. Sommers C. DeMarco D. R. Varkey S. Rhoden K. (2014). Detection of Shiga toxin-producing *Escherichia coli* (STEC) O157:H7, O26, O45, O103, O111, O121, and O145, and Salmonella in retail raw ground beef using the DuPont™ BAX® system. Front. Cell Infect. Microbiol. 4:81 10.3389/fcimb.2014.00081 PMC4061970 24995164

